# Cross-cultural comparison of attitudes toward seeking professional psychological help : A Multinational Population-Based Study from 16 Arab Countries and 10,036 Individuals

**DOI:** 10.1192/j.eurpsy.2023.747

**Published:** 2023-07-19

**Authors:** M. Stambouli, F. Fekih Romdhane, A. Jaoua, F. Ghrissi, W. Cherif, M. Cheour

**Affiliations:** Psychiatry department E, Razi hospital, Manouba, Tunisia

## Abstract

**Introduction:**

There has been an increasing interest in people’s attitudes toward seeking psychological help. Although recent research has shown a rise in the number of people seeking help from psychological services, there is still a significant number who choose not to see a mental health specialist.

**Objectives:**

The aim of the current study was to examine the attitudes toward help-seeking psychological help among Arab population and to investigate factors related to these attitudes in the whole sample.

**Methods:**

We carried out a multinational cross-sectional study using online self-administered surveys in the Arabic language from June to November 2021 across 16 Arab countries.The Community Attitudes toward the Mentally Ill scale,the Mental Health Knowledge Schedule scale and the Attitudes Toward Seeking Professional Psychological Help Scale-Short Form were administered to participants from the general public.

**Results:**

The study sample was predominantly female (77%), married (41%), educated (89% with tertiary education), living in urban areas (85%), with a mean age of 29.6 ± 10.8 years.

Participants tended to have a higher preference to seek help from a psychologist or a psychiatrist (85.7%) and primary care physicians (80.7%).We also found that family members represented a preferred source of help in 80.4% of the cases.

In bivariate analyses, help-seeking attitudes positively correlated with attitudes (r=.265) and knowledge (r=.121). Besides, multivariate regression analyses revealed that being female, older, having higher knowledge and more positive attitudes toward mental illness, and endorsing biomedical and psychosocial causations were associated with more favorable help-seeking attitudes; whereas having a family psychiatric history and endorsing religious/supernatural causations were associated with more negative help-seeking attitudes.

**Conclusions:**

Attitudes toward seeking professional psychological help are intricate. Determining factors associated with help-seeking attitudes may guide interventions in order to avoid delays in help-seeking.

**Disclosure of Interest:**

None Declared

